# 

**Published:** 1896-04

**Authors:** 


### TABLE OF CONTENTS.

ORIGINAL COMMUNICATIONS. PAGE

Suggestions on the Use of Electrical Osmosis in Dental Practice. By Peter Brown, L.D.S., Montreal, Canada . . . . . . . . . . . . . . . . . . . 197

Pyorrhœa Alveolaris. By Eugene S. Talbot . . . . . . . . . . . . . . 203

Oral Electricity and Vital Currents. By S. B. Palmer, M.D.S., Syracuse, N. Y. . . . . . . . . . . . . . . . . . . . . . . . . . . . . . 215

REPORTS OF SOCIETY MEETINGS.

American Dental Association . . . . . . . . . . . . . . . . . . . . . . . 220

The New York Institute of Stomatology . . . . . . . . . . . . . . . . . 235

EDITORIAL.

Duplication and Reduplication of Papers . . . . . . . . . . . . . . . . . 250

An Apology to the Editor of the *Digest* . . . . . . . . . . . . . . . . . 252

Crown Company beaten on Bridge Suit . . . . . . . . . . . . . . . . . . 253

BIBLIOGRAPHY . . . . . . . . . . . . . . . . . . . . . . . . . . . . . . 254

OBITUARY.

William H. Dwinelle, A.M., M.D., D.D.S. . . . . . . . . . . . . . . . . 256

CURRENT NEWS.

Northeastern Dental Association . . . . . . . . . . . . . . . . . . . . . 258

Woman's Dental Association . . . . . . . . . . . . . . . . . . . . . . . 259

Harvard Odontological Society . . . . . . . . . . . . . . . . . . . . . . 260

